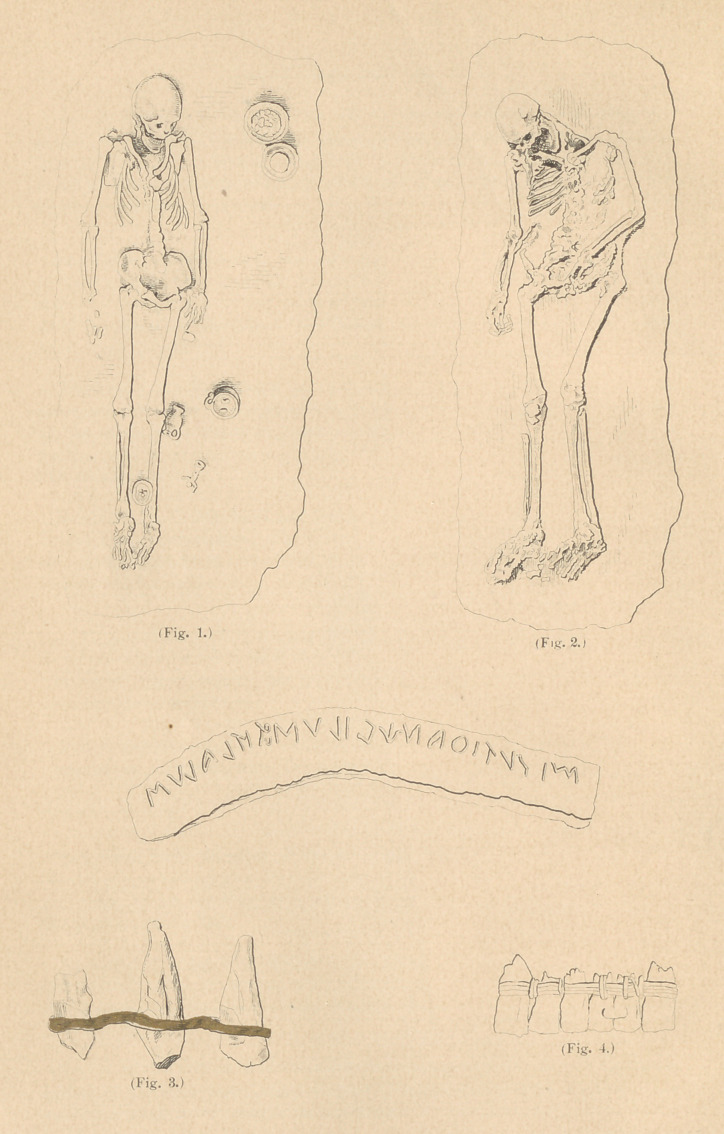# Further Evidences of Prehistoric Dentistry

**Published:** 1886-02

**Authors:** J. G. Van Marter

**Affiliations:** Rome, Italy


### Independent Practitioner.

Vol. VII. February, 1886. No. 2.

### Original Communications.

### FURTHER EVIDENCES OF PREHISTORIC DENTISTRY.

BY J. G. VAN MARTER, A. B., D. D. S., ROME, ITALY.

Since my communication of last year, I have pursued my researches after reliable traces of early dentistry. I especially tried to find some authentic case of pre-Roman tooth filling. The noble Princess, referred to in my former article on this subject, assisted me to see the teeth which she was sure were Etruscan teeth filled with gold. In the library of the Barberini Palace, in this city, most carefully guarded by lock and key and screw, I found this treasure-trove. Viewed under glass, this case might easily deceive the unprofessional eye, for it was thickly covered with the debris of ages. It took a great deal of persuasion to induce the polite and careful librarian to allow me to remove enough of the dust of centuries (he said it was sacred dust of three thousand years ago) to see what the Etruscan relic really was. It proved to be four natural teeth—two superior central incisors, lateral and cuspid—banded together with pure gold bands, and attached to adjoining teeth precisely like those described by me formerly. This case belongs to the same period as those I found at Corneto, and in workmanship was so nearly identical that it might have been made by the same dentist. It was taken from an Etruscan tomb at Palestrini, near Rome, with numerous fine specimens of gold and bronze-work. As a specimen of early tooth filling, this case is an utter failure, for the gold was *on* and *around,* and not in the teeth. Likewise, the gold in the mummy teeth, in the museum at Naples, reported to me by authorities, has vanished, and is not even *on* nor *around* the teeth.

Undeterred by these disastrous failures to find gold in the teeth, I proceeded to the Vatican, and by the kind intervention of Mgr. Cataldi, was freely permitted to make a careful search through the vast collection of Etruscan relics and treasures, but found no trace of prehistoric teeth or dental work to reward my patience. Private collections have been visited also, with like result.

The most extensive collections of Etruscan remains, skulls and teeth, in Italy, so far as I know, is to be found in the Museum of Bologna, which place I visited in October last, for the purpose of studying this most extensive and rare treasure-house.

It gives me pleasure to be able to send herewith several photographs of some of the best preserved remains in Etruscan tombs, which were taken on the spot. (See Figs. 1 and 2.) In the photo marked No. 1 the teeth were exceedingly fine in form and preservation. In No. 2 the teeth were equally fine, but twenty-eight in number, with no trace or sign of there ever having been thirty-two. In three other skulls I noted the same want of the third molars. Evidently they were never developed. It is worthy of note that, in the comparatively few remains of prehistoric skulls in the above collection, there should be such a proportion of those in which the third molar does not appear. About one-fourth of the third molars were wanting. What then becomes of the theory that the wisdom teeth are becoming rudimentary and disappearing? Perhaps they disappeared once before, and reappeared again in an age of wisdom, but are now fading away, marking a decadence in that dental evidence of sage understanding.

In one case, of which I could not get a photograph, there were the remains of a mother and child lying side by side. The child had the first set of teeth complete, but there was a decided malarticulation. Whether this malformation of the inferior maxillary existed prior to death, or whether the slow accumulation of the debris of ages, or filling in of the tomb, has crowded the lower maxilla into the present malposition, I cannot say for certain. I incline, however, to the opinion, from a careful study of the case, that there must have been a malformation of the inferior maxillary prior to death. The mother, lying by the side of her child, had evidently in life lost all the inferior teeth except the two cuspids, and the alveolar process had absorbed away, and looked precisely as we see such cases nowadays, where the poor unfortunate has worn a plate to supply the defect. In this case the superior maxillary and teeth were wanting. In none of the teeth in this collection did I discover any appearance of caries. All the teeth that were in the skulls, or parts of skulls, were sound, and splendid in form and size. Judging from the case of the mother above described, some of the missing teeth in some of the skulls may have been extracted to relieve pain, for want of proper treatment.

By the side of these Etruscan remains lie one Umbrian skeleton and ten skulls. The teeth in these skulls were very fine. Although these Umbrians preceded the Etruscan race, their heads were of the same type and size, with nothing particular to remark about them. There was an extensive collection of Umbrian domestic articles, such as safety pins, various ornaments, hammers, bridle bits for horses, the same form as that used in these modern times, and a vast number of other things. I am assured by those who have studied the subject deeply, that these Umbrians understood the principles and use of the telephone and tramway.

I must not omit to mention that in this Bologna collection there is an Egyptian mummy, in which the superior left central and lateral incisors were decayed away, and the right central about half broken down with caries. Evidently *some* Egyptians were acquainted with caries and grief. There were also numerous specimens of early Gallic teeth in this museum, from the Province of Succa, in various stages of malformation, decay and irregularity.

The most recently opened and the oldest Etruscan tomb yet discovered in Italy was lately excavated at Capadimonti, near the Lake of Bolsena. The entire contents of this tomb, including three teeth bound together with a band of pure gold, gold spiral rings for the side hair, silver finger ring, necklace of amber and glass, arm band, bronzes, vases, etc., etc., I take pleasure in sending you by first express. The part of this find of interest to our profession is the three teeth, a drawing of which I send you herewith. (See Fig. 3.) This tomb belongs to the VIth Century, B. C., or about one hundred years prior to the dates of the oldest partial denture which I sent you last year.

The manner of banding the teeth together is more primitive than the Corneto dental specimen, and marks a distinctly earlier stage of pre-Roman dentistry. There is nothing to indicate that these three teeth were attached to any adjoining teeth, and we are left to conjecture whether they were loose natural teeth, supported by the gold band, or if the cuspid were transplanted and held in position by the gold band around the lateral and bicuspid. It is not at all improbable that the cuspid may have been a transplanted tooth, for we are sure that in those early days they had very clever surgeons, and slaves were made to serve their lords and masters in any capacity, from building grand temples and monuments to supplying teeth for transplantation. Certainly the spaces between these teeth are wide enough to satisfy the most rabid dental separatists, and the position of the teeth does not indicate that perfect regularity and symmetry were the invariable rule, even in those early days. This is significant, when we consider that the former owner of these teeth was evidently a lady of distinction, judging from the ornaments and the contents of the tomb. At least this specimen of early Etruscan dental work is of interest to us, as the oldest yet found in Italy, and as supplying one of the missing links of the dental chain we are endeavoring to trace back to the beginning of our profession.

Still older than this Etruscan specimen, I am assured by competent authorities, is the specimen of Phœnecian dentistry, a sketch of which I send you herewith. (See Fig. 4.)

This example of early dentistry is described by M. Ernest Renan, in his work, entitled "Mission de Phénicie e le Campagne de Sidon," page 472, Paris, 1864, as follows:

"But that which was most interesting was the upper portion of a woman's jaw, showing the two superior cuspids and four incisors, united by a gold thread. Two of these incisors seemed to have belonged to another person, and to have been placed here in order to replace the missing ones. This piece, which was found in one of the most ancient vaults, proves that the art of dentistry was pretty far advanced at Sidon, and also proves that the earth scurvy (*scorbut de terre*), so commonly seen nowadays in Sidon, existed already in those ancient times."

I am informed that this piece of Phœnecian dentistry may be seen in the Museum of the Louvre, Paris.

It will be observed that this Phœnecian example of dental handiwork marks a still earlier period in the art of dentistry than the two other styles which I have already described.

We have then the illustration of the Etruscan, 500 years B. C., which I sent you last year; the 600 years B. C., which I now send you, and the Phœnecian.

It is certain that dentistry must have been extensively practiced in the early history of the world, and that gold must have been used largely; otherwise the early Greek and Roman legislators would not have mentioned the matter in the celebrated laws of the twelve tables. Law 5th, *de Jure Sacrorum*, is as follows: *Quoi auro dentes vincti sient in cum ollo sepelire, se frande esto.* "If any-one's teeth have been bound togethr with gold, it shall not be un-lawful to bury him with it" (the gold).

These twelve tables of the laws date from 447 B. C. The Romans took their laws from the celebrated Greek Solon, which takes us back to 625 B. C. The early Romans learned their dentistry from the Etruscans, and evidently the Etruscans and Greeks learned all they knew of dental art and science from the Egyptians; hence we must go back to the " mother of the arts and sciences" as the fountain head.

## Figures and Tables

**Figure f1:**